# CD83 Regulates the Immune Responses in Inflammatory Disorders

**DOI:** 10.3390/ijms24032831

**Published:** 2023-02-01

**Authors:** Bushra Riaz, S. M. Shamsul Islam, Hye Myung Ryu, Seonghyang Sohn

**Affiliations:** 1 Department of Biomedical Science, Ajou University School of Medicine, Suwon 16499, Republic of Korea; 2 Department of Microbiology, Ajou University School of Medicine, Suwon 16499, Republic of Korea

**Keywords:** inflammatory disease, CD83, dendritic cell, herpes simplex virus

## Abstract

Activating the immune system plays an important role in maintaining physiological homeostasis and defending the body against harmful infections. However, abnormalities in the immune response can lead to various immunopathological responses and severe inflammation. The activation of dendritic cells (DCs) can influence immunological responses by promoting the differentiation of T cells into various functional subtypes crucial for the eradication of pathogens. CD83 is a molecule known to be expressed on mature DCs, activated B cells, and T cells. Two isotypes of CD83, a membrane-bound form and a soluble form, are subjects of extensive scientific research. It has been suggested that CD83 is not only a ubiquitous co-stimulatory molecule but also a crucial player in monitoring and resolving inflammatory reactions. Although CD83 has been involved in immunological responses, its functions in autoimmune diseases and effects on pathogen immune evasion remain unclear. Herein, we outline current immunological findings and the proposed function of CD83 in inflammatory disorders.

## 1. Introduction

Dendritic cells (DCs), which are considered potent antigen-presenting cells (APCs), act as vital mediators of immunological balance that can directly identify microbial antigens through the environmental signals or by reacting to factors released by several immune cells to regulate immune responses [1]. Numerous mechanisms could be involved in how DCs regulate immunological tolerance, such as apoptosis of DCs to prevent the accumulation of DCs and preserve self-tolerance. In addition, different subsets of DCs can functionally promote the development of T cells [2]. The cellular activation of DCs and T cells is imperative for the initiation of adaptive cell-mediated immunity. Within the lymph node, T cells receive signals from antigen-bearing DCs. T cell–DC contact is a highly regulated event that determines the timing, signal strength, and inflammatory environment of the responding T cells. The fate of T cells is determined by their integration with antigen-bearing DCs and their ability to process signals that activate T cells [3].

When an antigen is captured by DCs, it undergoes a maturation process. Mature (mat) DCs have developed the capability to differentiate B cells, naïve T cells, and natural-killer (NK) cells. Fully activated DCs are capable of accumulating processed peptides and give an additional surface expression of the major histocompatibility complex (MHC) and costimulatory molecules CD40, CD80, CD83, and CD86 [4]. Among costimulatory molecules, CD83 has the function of promoting the expression of other activation markers such as CD86 and the MHC class II [5]. A variety of immune cells, including B cells, thymus-epithelial cells (TECs), T cells, DCs, and neutrophils, are known to express the CD83 molecule. CD83 is essential for the activation of T cells that regulate peripheral immune responses [6]. CD83 is one of the markers of matDCs, as CD83 is highly expressed during DC maturation [7]. Activation, migration, and maturation of DCs initiate protective immunity and immune tolerance to the host, but dysregulated trafficking of DCs leads to disturbance of immune homeostasis and can cause abnormal inflammatory responses due to impaired tolerance [8]. This article reviews the relationship between the DC activation marker CD83 and its therapeutics in inflammatory disorders.

## 2. Characteristics of CD83

In this section, we provide a brief overview of CD83 regarding its structural features, immunological function, and signaling pathways, with an emphasis on how crucial it is for triggering and resolving immune responses.

### 2.1. Structural Features of CD83

CD83 is a glycosylated type-1 transmembrane glycoprotein and a member of the immunoglobulin (Ig) superfamily found predominantly on DCs surface, cutaneous Langerhans (LC) cells, and circulating DCs for recognizing the matDCs [9,10,11,12]. The human CD83 (hCD83) protein consists of 205 amino acids, whereas the mouse protein has 196 amino acids. Overall, human CD83 and mouse CD83 share 63% amino acid similarity. In the extracellular domain, 10 amino acids were deleted in the mouse CD83 compared to human CD83, showing a homology of 56% between the two [13]. CD83 can form homodimers in a prokaryotic expression system [14]. A protein crystal structure study has found remarkable structural similarities between B7 family members and CD83 [15]. It has been hypothesized that CD83 might exert its immunological function through homotypic or heterotypic associations with a ligand. The extracellular domain of CD83 is folded into a V-set Ig domain. Human CD83 contains five cysteine residues in the same domain, creating two pairs of disulfide linkages. This information was also obtained by analyzing its protein crystal structure [14,15,16]. Although mouse CD83 gene structure has already been extensively studied, the promoter region of human CD83 has just recently been discovered [17]. To date, two isoforms of CD83 have been identified, including a full-length membrane-bound (mb) CD83 and a soluble (s) CD83 comprising only the extracellular domain [18].

### 2.2. Immunological Function of CD83

**Figure 1:**
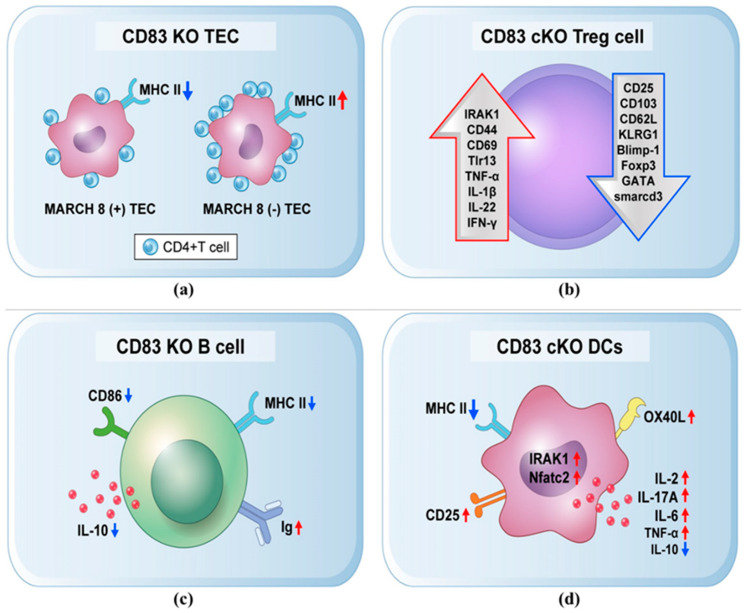
CD83 knockout (KO) mice model displayed abnormal tolerance. (**a**) CD83 KO TEC showed a strongly decreased number of CD4+ T cells and deletion of MARCH 8 in CD83KO TEC restored CD4+ T cells. (**b**) In CD83 cKO mice, CD83 is only ablated in Treg cells, and expression of Treg-specific maturation biomarkers (CD25, CD103, CD62L, or KLRG1) and transcriptional factors (Blimp-1, Foxp3, GATA, and Smarcd 3) are reduced. Signaling pathway (IRAK1), inflammatory cytokines (TNF-α, IL-1β, IFN-γ, IL-22), and other biomarkers (CD44, CD69, Tlr13) were present at high levels. (**c**) CD83 KO B cells have reduced IL-10, MHC II, CD86 expression levels, and increased Ig levels. (**d**) CD83 KO DCs expressed higher levels of costimulatory molecules (OX40L, CD25) and transcription factors (IRAK1, Nfatc2) and significantly more cytokines (IL-2, IL-17A, IL-6, TNF-α) were secreted. Overall, CD83 expression is essential for ongoing immunological responses. TECs, thymic-epithelial cells; MARCH 8, membrane-associated RING-CH-type protein; KLRG1, killer-cell lectin-like-receptor subfamily-G-member-1; IRAK1, Interleukin 1 Receptor Associated Kinase 1; Tlr, Toll-like receptor; TNF-α, tumor-necrosis factor-α; IL-1β, interleukin-1-beta; IFN-γ, interferon gamma MHC, major-histocompatibility complex; Ig, immunoglobulin; IL, interleukin.

Among distinct cell types, CD83 serves a variety of functions. CD83 molecule can modulate immunological responses through activated T cells, TECs, regulatory T cells (Treg), B cells, and DCs. For instance, in the thymus, CD83 expression is another regulating factor necessary for the formation of CD4+ T cells. However, CD83 reduction on TECs can impair thymic CD4+ T cells. Rohrscheidt et al. [19] conducted a CD83 knockout (KO) animal study and revealed that mbCD83 is essential for T cell activation in the thymus, and CD83 KO mice strongly decreased numbers of CD4+ T cells by reducing the MHC II expression on the periphery. Membrane-associated RING-CH (MARCH) 8, an E3 ubiquitin-ligase, is regulated through CD83, which then in turn controls MHCII trafficking on TECs. In CD83 KO mice, the deletion of MARCH 8 can restore CD4+ T cell selection to the control level by enhancing MHC II expression (Figure 1a). As a result, CD83 and coordinated control of MHC II expression on the surface of TECs via MARCH 8 is crucial for the selection of CD4+ T cells. This functionalized pathway is critical for selective thymic-positive CD4+ T cell development [19,20]. CD83 expressions are not confined to thymic CD4+ T cell activation because CD83 is also expressed in various T cell sub-populations, suggesting that CD83 has a unique function in controlling T cell development.

Strong CD83 expression is also observed in murine Treg cells. Murine CD83+ CD4+ T cells can upregulate various Treg markers (CTLA-4, CD25, GITR, NRP-1, or Helios) upon activation [21]. Human CD4+ T cells can also enhance CD83 expression via T cells receptor (TCR) stimulation. Remarkably, TCR stimulation of murine and human CD4+ T cells with transforming growth-factor-β (TGF-β) can stabilize CD83 expression [22]. Furthermore, the expression level of intrinsic CD83 on Treg cells has been observed in cKO mice, whereas CD83 is exclusively ablated in Foxp3+ Tregs. Consequently, there has been a decrease in the expression of Treg-specific maturation biomarkers (CD25, CD103, CD62L, or KLRG1) as well as transcriptional factors (Blimp-1, Foxp3, GATA, and Smarcd 3) while inducing signaling pathway (IRAK1). Other biomarkers (CD44, CD69, Tlr13) and inflammatory cytokines (TNF-α, IL-1β, IL-22, IFN-γ) were also present at higher levels (Figure 1b) and exhibited an impaired tolerance in Treg CD83 cKO mice [23,24].

Additionally, CD83 has been identified as a biomarker for B cells known to have powerful CD83 activity. When B cell receptors, CD40, and TLRs are activated, CD83 expression is increased to control B cell activity. Polyclonal compounds such as CD40L [25], anti-CD40, lipopolysaccharide (LPS), and anti-IgM [26] can stimulate murine B cells to produce CD83 on their cell surface. It has been demonstrated that phorbol myristate acetate (PMA) [27], CpG [28], and pathogenic bacteria [29] can stimulate human B-lymphocytes to increase CD83 expression. Analyzing the functional impact of CD83 on B cells is challenging since CD83 KO mice have significantly fewer peripheral CD4+ T cells. However, Prazma et al. [26] generated CD83 KO mice and revealed that CD83 activity is only crucial for B cell longevity. In contrast, Kretschmer et al. [30] developed CD83 KO mice that express less CD83 expression. These mice display reduced MHC II and CD86 expressions along with decreased IL-10 and increased Ig levels in supernatants of LPS-stimulated B cells as compared to CD83 transgenic (CD83tg) mice, which drastically show the opposite function (Figure 1c). Taken together, these results suggest that CD83, after being expressed on the B cells surface upon activation, can help regulate B cell activity.

CD83 exposure in DCs is essential for DC activity. For example, when CD83 activation is downregulated using RNA interference (RNAi) in human DCs, established T cells show reduced IFN-gamma secretion, tumor antigen-specific CD8+ T lymphocyte proliferation, and allogeneic T cell proliferative capacity [31]. In this regard, CD4+ T cells that grow in CD83-mutated animals do not respond to allogeneic stimulation because of a changed pattern of cytokines expression that is associated with elevated releasing of IL-4 and IL-10 and reduced secretion of IL-2 [32]. Moreover, Bates et al. [33] conducted a CD83 KO colitis mice model and found that CD83 deletion on DCs exacerbates disease symptoms. Furthermore, CD83 KO bone-marrow-derived DCs (BMDCs) did not show any difference in CD86 and MHCII expression after CD83 deletion. A later study reported that decreased expression of CD86 and MHC-II in BMDCs was associated with CD83 depletion [34]. Interestingly, other researchers found that MHCII and CD86 surface expressions on BMDCs were stabilized either by the transmembrane region of CD83 or by opposing the effects of MARCH 1 [5]. Surface expressions of CD86 and MHCII provide strong evidence that CD83 expressions seen on DCs also affect T cell stimulation. It has been suggested that increasing surface MHCII expressions as seen in the DCs of MARCH 1 KO mice can disturb DC homeostasis. In this regard, MARCH 1 KO DCs have markedly diminished IL-12 release with CD4+ T cells modulatory capacity [35]. Additional findings indicated that DC-specific CD83 KO mice expressed higher levels of costimulatory molecules (OX40L, CD25) and transcription factors (IRAK1, Nfatc2), and significantly more cytokines (IL-2, IL-17A, IL-6, TNF- α) were secreted (Figure 1d). This improved the activation of T cell response and impaired the inhibitory activity of Treg cells [36]. We also noticed that CD83 homotypic binding could affect DC cytokine production functionally, lessen levels of DC cytokines such as IL-12p40 and MCP-1, and be known to significantly promote mucosal inflammation [33]. When considered collectively, CD83 deletion on DCs can lead to increased immunological responses by dysregulating tolerance pathways, thus compromising the clearance of inflammation, despite MHCII and CD86 surface expression levels being reduced.

### 2.3. Soluble CD83 versus Membrane-Bound CD83-Signaling Pathway

Although CD83 is considered a functional adhesion receptor that regulates cellular immunity, the ligand or receptor pattern of CD83 has remained evasive for a prolonged time. Higher expression of CD83 has been found on activated neutrophils, Treg cells, macrophages, and activated B and T lymphocytes [9].

sCD83, an extracellular domain of mbCD83, displays an immune-suppressive characteristic that can be released from activated DCs and B cells [37]. Various studies have demonstrated sCD83’s binding pattern, mainly focusing on its interaction with putative receptors [38,39,40,41]. Interestingly, myeloid differentiation factor-2 (MD-2), a coreceptor of Toll-like receptor (TLR) 4, can act as a binding partner for sCD83 through TLR4/MD-2 complex in monocytes depleted of IRAK-1 protein [42]. sCD83 inhibited acute rejection in rat transplantation experiments by increasing the expression of TGF-β, activating the IDO immunosuppressive pathway, and increasing Treg cells [43]. In addition, CD83/TLR4 axis can lead to Treg induction by increasing Krüppel-like factor 10 (Klf10/TIEG-1) expression, a transcription factor used for modulating TGF-β expression [44]. Recently, Lin et al. [45] proposed that the sCD83 signaling pathway demonstrated the inhibitory activity of sCD83 that counteracts aberrant T cell activation. This inhibitory effect of the sCD83 is caused by reduced DC-T cell contact and the formation of DC-T synapses due to the loss of MHC-II and GTP-binding ability of Rab1a. Roco2-kinase family (LRRK2) might help activate Rab-1 and control the F-actin arrangement. sCD83 interrupts the LRRK2/F–actin complex in DCs by Rab1a. Thus, it has a vital role in developing immunosuppressive agents for autoimmune diseases. Another publication has shown that sCD83 administration can suppress Bcl2L12, an apoptosis inhibitor that causes allergic rhinitis, by binding CD154 (CD40L) to Th2 to enhance the transcription factor IRF1 expression. Therefore, sCD83 has been used to treat an allergic rhinitis model to improve apparent symptoms of the disease [46]. The upregulation of prostaglandin E2 (PGE2) level induced by NF-κB in CD83-affected monocytes suppresses T cell proliferation and releases IFN-γ and IL-2 via T cells, consequently modulating T cell immunity [47].

Numerous cell types that have been discovered to be able to bind sCD83 also express mbCD83. For example, for human T cells to bind to sCD83, they have to be activated first with agonistic anti-CD3/CD28 antibodies. This can also induce the production of mbCD83 expression. Furthermore, the cytoplasmic tail of CD83 generally lacks a consensus signaling motif. Thus, its homotypic association with other proteins might serve as a scaffold to encourage the enlisting of many other proteins as signal transducers. Both sCD83 and mbCD83 are integrated into the homotypic interaction because of their immune-regulatory and immunomodulatory role, respectively. Immunological reactions are more susceptible to malfunction when one interacting partner is missing. In summary, mbCD83 plays a role similar to that of sCD83 in part to enhance the ongoing immunological response.

## 3. CD83 Molecule and Herpes Simplex Virus

Viruses are tiny infectious agents that are able to multiply in living cells and are transmitted by infection. Viruses and immunological responses are in constant competition with each other. Viruses use a variety of processes to evade immune recognition and create more effective and widespread infections. DCs are necessary for initiating an immunological response against viruses. DCs play a vital role in how viruses enter cells, disseminate, survive, and transmit to target cells. To accomplish this, viruses change the function and expression level of receptors, signaling pathways, and antiviral compounds; obstruct trafficking pathways; or even interact with other cell types [48]. The fact that multiple viruses target the CD83 surface molecule was a highly intriguing discovery. Different viruses can affect CD83 expression levels both directly and indirectly.

**Figure 2:**
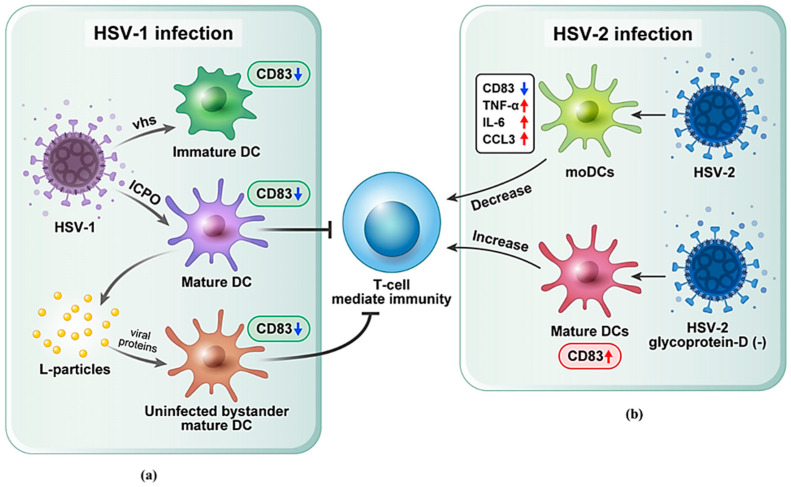
Correlation between CD83 and HSV. (**a**) HSV-1 proteins vhs and ICP0 degraded the CD83 on iDC and matDC, respectively. Uninfected-bystander matDCs showed a significant CD83 reduction because L-particles are released from infected matDCs. Uninfected bystander matDCs and infected matDCs prevent T cell activation. (**b**) HSV-2 infection on moDCs reduced CD83 levels and enhanced cytokine and protein levels (TNF-α, IL-6, CCL3), further attenuating the T cell responses. When glycoprotein-D deleted HSV-2 virus is exposed to DCs, T cell stimulation is enhanced. HSV, Herpes simplex virus; vhs, host-shutoff protein; ICPO, infected-cell protein-0; moDCs, monocyte-derived-DCs; TNF-α, tumor-necrosis factor-α; IL-6, interleukin-6; CCL3, Chemokine ligand 3.

Herpesviridae family members are involved in DC activation. One of the prototypical members of the α-herpesviridae is the herpes simplex virus (HSV). HSV has a specific ability to infect both mature (mat) and immature (i) DCs, impairing the function of infected DCs. DCs that expose HSV entrance receptors HVEM (Hve-A) and nectin-2 (Hve-B) are highly susceptible to HSV infection [49,50]. DC-SIGN, a c-type lectin exposed by DCs, is another important receptor essential for HSV transmission and infectivity [51]. Furthermore, HSV-1-infected iDCs inhibit their maturation process, including CD83 upregulation [50]. A protein of HSV-1 known as the virion host shutoff (vhs) protein is essential for preventing DC maturation. In these circumstances, LPS-stimulated DCs derived from human PBMCs with such vhs knockout mutations retained the potential to upregulate the CD83 marker to significant levels. It appears that vhs protein is critical for blocking LPS-induced iDCs maturation. However, CD83 degradation on matDCs does not depend on vhs [52]. Infected cell protein 0 (ICP0) is another important immediate-early (IE) protein of HSV-1. ICP0 is necessary and sufficient to cause CD83 degradation on matDCs derived from human PBMCs via ubiquitin-independent cellular proteasome [53]. Moreover, HSV-1 infection of matDCs can cause a notable CD83 downregulation, which can reduce their ability to activate T cell-mediated immunity [54]. Furthermore, not only HSV-1-infected matDCs but also uninfected bystander cells display a substantial CD83 reduction. This is due to L-particles released during the replication cycle of HSV-1. These L-particles are not infectious due to the loss of viral DNA and capsid. However, they contain several cellular factors and viral proteins. These functional proteins can be transferred from infected matDCs of human PBMCs to uninfected bystander matDCs and thus downregulate CD83 expression (Figure 2a) [55]. Although the significant role of L-particles in exposing and regulating CD83 expression is not precisely understood yet, Birzer et al. [56] performed mass spectrometry of L-particles and found that the concentration of ICP0 in L-particles was lower than that of other proteins such as ICP6, UL42, gB, gD, and ICP4. Additionally, L-particles can influence DC function by altering CD83 expression and preventing T cell activation. Moreover, HSV-1 targets CD83 for destruction in infected matDCs isolated from human PBMCs, which can result in significant decreases in both intracellular and surface-exposed CD83 protein levels [55,57]. Since CD83 mRNA level is not decreased, it cannot be assumed that CD83 expression is reduced due to the inhibition of mRNA synthesis. Thus, CD83 down-modulation must be triggered by a post-transcriptional event. It has been shown that matDCs infected with HSV-1 have a significantly decreased ability to stimulate T cells [54]. However, the specific molecular pathway of CD83 degradation caused by HSV-1 is still inexplicable. It is noteworthy that HSV-2 can modify the CD83 expression on DCs. As an illustration, Peretti et al. [58] have infected monocyte-derived-DCs (moDCs) isolated from rhesus macaques with HSV-2. This HSV-2 infected moDCs showed decreased levels of CD83 expression and other co-stimulatory molecules with increased levels of cytokines and proteins such as TNF-α, IL-6, and CCL3, further enhancing weaker T cell responses and other immunodeficiencies to viral infection. Moreover, HSV-2 can stop DC maturation and promote proteasome-dependent CD83 reduction on matDCs [59,60]. However, when glycoprotein D, a protein required by HSV-2 to penetrate host cells, was deleted from HSV-2 virus and when DCs were exposed to this modified virus, their ability to migrate and activate naive T cells was enhanced, leading to noticeably less infection and pathological infection (Figure 2b) [61].

Consequently, HSV targets CD83 activity directly or indirectly, emphasizing the critical function of viral proteins and CD83 molecules during the activation of immunological responses and participation in the treatment of severe viral infections.

## 4. CD83 and Inflammatory Disorders

An intricate collection of diseases known as inflammatory disorders are caused by immune system dysfunction. These inflammatory disorders have an undesirable impact on life quality. They are hard to treat. Numerous reports of CD83 involvement in inflammatory processes show its critical functions in immunological responses (Table 1). Moreover, therapeutic approaches that target CD83 and its ligands to inhibit inflammatory immunological responses have emerged as a consequence of our increased understanding of the expression and function of CD83 (Table 2). Below, we detail the involvement of CD83 in several inflammatory disorders.

### 4.1. Inflammatory Bowel Disease

Inflammatory bowel disease (IBD) and its subtypes ulcerative colitis (UC) and Crohn’s disease (CD) epitomize the intestinal inflammation in which immunosuppressive and immunostimulatory mechanisms are disturbed in the gastrointestinal (GI) tract [62,63]. Recently, Yu et al. [64] found that intestinal epithelial cells (IECs) display CD83 and aid in Treg cell activation in the intestine that can specify immune tolerance in the GI tract [21]. However, an imbalance in the ratio of T effector cells to Treg cells might have a role in the etiology of IBD [65]. IECs of CD83 KO mice are unable to stimulate Treg cells in tissues of the intestine, indicating CD83 is essential for Treg maturation in the intestine [64]. Likewise, the colon mucosa of IBD had T cell areas of lymphoid follicles that contained CD83+ cells, although there were fewer CD83+ cells outside of these follicles [66]. By using a murine transferring colitis model, Kreiser et al. [21] established that CD83+ T cells have the capacity to decrease colitis symptoms in vivo. Growing evidence has shown that intestinal DCs induce immunological tolerance in the gut through IDO expression, which can further enhance gut immune stability by restricting inflammatory activities [67,68]. According to one publication, IDO is one of several immune-suppressive mediators expressed by Treg cells [69]. Treatment with sCD83 can increase the expression level of IDO. In addition, sCD83 can decrease the expression of proinflammatory cytokines, inhibit the infiltration of granulocytes and macrophages to the colonic tissue, and reduce the mortality rate of a colitis experimental mouse model [70]. Extensive research has shown that in the intestinal lamina propria, DCs can organize a vast network to control the mucosal immune responses. Changes in DC function might increase the risk of IBD. A reduction in CD83 DCs levels can worsen the inflammation in the colitis model. However, CD83 DCs overexpression on the mucosal surface can protect against colitis, demonstrating the functional aspect of CD83 upon DCs in immunological homeostasis [33]. Parallel to this, it has been discovered that *Tetragenococcus halophilus*, a lactic acid-producing bacterium, can suppress DSS-induced colitis in mice exhibiting increased CD83 molecule expression [71]. In the same model, this bacterium reduced CD83 expression along with a decrease in CD8+NK1.1+ cells and IL-1β levels. CD83 DCs are more common in patients with CD and acute inflammation [72]. We have clarified that CD83 molecule expressions are increased in colonic tissue samples from CD patients as a function of DC-induced increased expressions of various lymphoid chemokines at inflammatory sites in CD patients [73]. These important findings imply that CD83 is a key checkpoint that is upregulated at sites of intestinal inflammation. Strategies that modulate CD83 could be another candidate in the process of establishing numerous new potential therapies to alleviate clinical symptoms.

### 4.2. Systemic Lupus Erythematosus

Systemic lupus erythematosus (SLE) is an inflammatory, chronic, and autoimmune disorder that often manifests as irregularities in the immune activation system among several organs. However, there is an imbalance in the adaptive immune system, including autoantibodies, T-helper subgroups (Th1/Th17), Treg and B cells [74,75], and elements of innate immunity, including DCs and complements, which are all included in SLE development [76]. DCs are associated with abnormal functionality in SLE. For instance, the myeloid (m) DC marker selectively displays distinct functional and phenotypic alterations on DCs from SLE patients and elevated abnormal T cell function. It also exhibits aberrant reactions to maturational stimuli [77]. However, in another publication, reduced expression levels of mDCs and CD83 seen in SLE patients might contribute to a higher infection prevalence [78]. In addition, CD83 considered a classical biomarker of matDCs was only increased in monocytes of SLE patients. It did not show a significant alteration in CD83 expression on mDCs or on plasmacytoid (p) DCs [79]. Moreover, Treg cells are required for the sustenance of immune homeostasis, and thus a reduction in Treg cells causes SLE [72,80]. The latest study has revealed that CD83 derived from epithelial cells has a crucial role in renovating immune balance by stimulating Treg cell activation [73]. An additional experiment has established that SLE is caused by variations in the composition of microparticles created by cell-membrane budding during or after apoptosis [81,82]. These microparticles extracted from the plasma of SLE patients show elevated CD83 expression and the production of proinflammatory cytokines such as interleukin-6 (IL-6), TNF, and interferon-α by pDCs and mDCs, leading to autoimmune response in SLE patients [83]. In vitro, high-mobility group box protein-1 (HMGB1) is tightly coupled to nucleosomes expelled by late apoptotic cells. This protein has been detected in the plasma of SLE patients. It can cause the upregulation of CD83 markers, thereby breaking the immunological balance against nuclear antigens through their proinflammatory activities on distinct cell types, predominantly DCs and macrophages [84]. Mesenchymal stem cells (MSCs) generated from the umbilical cord (UC-MSCs) can dramatically stimulate the DC subset (CD1c+) in SLE patients and upregulate tolerogenic features by reducing the expression of CD83 and other co-stimulatory markers and decreasing TNF-α production while still keeping IL-10 production. Therefore, UC-MSC-produced CD1c+DCs might ameliorate immune dysfunction and preserve immunological homeostasis, which might contribute to the therapeutic advantages of SLE [85]. The function of human leukocyte antigen (HLA) g in immunity has been identified, exerting a possible role in SLE [86,87]. MatCD83+ DCs and monocytes from SLE patients show reduced expressions and functions of HLA g that can cause an immunological imbalance in this autoimmune environment [88]. Another study has indicated a well-established link between complement component-1q (C1q) and various autoimmune disorders, including SLE. C1q genetic deficiency is a significant risk factor for SLE. In recent years, there has been extensive research performed on C1q’s function in controlling DCs differentiation and function. A C1q deficiency affects self-tolerance maintenance and dysregulates the differentiation of monocytes into DCs. C1q-cultured monocyte-DCs display a decrease in CD83+ frequency [89,90]. Furthermore, a transcriptome-wide association study (TWAS) has discovered a substantial correlation between CD83 and SLE, providing potential targets for SLE treatment [91]. Starke et al. [92] have proposed that human sCD83 treatment displayed a modulatory role in the development of autoantibodies involved in autoimmunity and obstruct autoimmunity in an SLE mice model. Shirley et al. [93] have described curcumin, a compound found in the spice turmeric, acting as an immuno-suppressant to decrease the expression level of CD83 by inhibiting the proliferation of CD4+ T cells and DC migration and limit levels of chemokines, resulting in a failed response of human DCs to immunological stimulants. Taken together, the above findings suggest that CD83 might be helpful in interfering with autoantibody-linked autoimmune diseases and autoimmunity in SLE.

**Table 1:** Involvement of CD83 in inflammatory disorders.

Diseases	Years	Study Design	Main Findings	Ref.
IBD	2006	Examined colon tissue samples of CD patients.	CD83 molecule expression increased in the colon tissue samples of CD patients.	[94]
	2015	Used lamina propria of CD83 knockdown mice that develop worsened colitis using dextran sodium sulfate (DSS).	In the colitis mice model, CD83 marker expression in DCs was downregulated and caused the inflammation to worsen.	[33]
	2020	Analyzed the colonic biopsy sample of human patients with CD and UC.	The prevalence and abundance of CD83 marker vary among IBD subtypes and are more common in patients with CD than UC	[95]
	2021	Tiny intestinal segments expunged from mice	IECs display CD83 and aid in Treg cell activation in the intestine. Imbalance in T-effector cells to Treg cells may have a role in the etiology of IBD.	[64]
	2022	Analyzed the PBL, IELs, splenocytes, lymph node (LN) cells, and distal colonic part of DSS-induced colitis mice.	Expression level of CD83 marker is significantly higher in DSS-colitis mice.	[71]
SLE	2008	Used blood samples of SLE patients	Myeloid DCs and CD83 expression level decrease and may explicate the infection in SLE patients.	[78]
	2008	Collected serum, PBMCs, and peritoneal macrophages from the blood of SLE	HMGB1 protein was found in the plasma of SLE patients which elevated CD83 expression and thus broke the immunological balance.	[84]
	2010	Used PBMCs from the blood of SLE patients	CD83 expression only increased in the monocytes of SLE patients.	[79]
	2016	Analyzed the microparticles isolated from SLE patients’ plasma	Microparticles enhance CD83 marker expression and induce autoimmunity in SLE patients.	[83]
	2022	Analyzed the PBL of SLE patients	TWAS identified a significant correlation between CD83 and SLE	[91]
AD	2002	Screened the biopsy skin sample and blood taken from AD patients	TSLP dramatically upregulates the expression of CD83 and some other DC markers and was predominantly expressed by epithelial cells, particularly keratinocytes obtained from AD patients	[96]
	2007	Collected skin biopsies sample from chronic AD patients	Expression level of the CD83 marker is higher in AD.	[97]
	2020	Screened serum IgE from AD patients	MoDC culture stimulated with TGase3 showed the higher expression of CD83 and other DC markers and play a major pathobiology role in AD.	[98]
	2021	Used tape strips from AD lesion and non-lesion skin	DC marker expression such as CD83 is increased in AD tissue.	[99]
BD	2019	Collected the PBL and peritoneal macrophages from BD mouse	A mature CD83 marker expression level is higher in BD patients.	[100]
	2021	Collected the PBL and peritoneal macrophages from BD mice model	Noise stress increased mature CD83 expression in BD mouse model.	[101]
MS	2008	Collected spleen samples from mice	Higher CD83 expression on naïve CD4+ T cells transduced them into T cells suppressed the effector stage of the paralytic EAE symptomatic model.	[24]
	2012	Examined PBMCs, monocytes, and T cells isolated from blood	Observe low levels of mature CD83 marker in mature and tolerogenic DCs isolated from MS patients.	[102]
	2013	Analyzed blood, PBMCs, and CSF of MS patients	Frequency of CD83+ B cells is higher in secondary progressive MS patients.	[103]
	2018	Analyzing the serum sample separated from MS patients	sCD83 expression level is higher in the serum of MS patients.	[104]
	2019	Prepared a transgenic mouse model, collected the blood, spleen, and lymph node from these transgenic mice	hCD83 regulates Treg phenotype in hCD83-BAC mice and recovers faster from EAE –related symptoms as compared to wild mice.	[105]
RA	2002	Analyzed the SF and synovial tissue of RA patients	CD83 expression levels are higher in ST and SF samples of RA patients.	[106]
	2006	Analyzing the SF and serum samples of RA patients	sCD83 level is much higher in the SF from RA patients.	[107]
	2008	Used the synovial tissue obtained from the inflamed joint of both RA and PsA patients	Low expression levels of mature CD83+ are observed in RA and PsA patients.	[108]
	2017	Plasma, SF, and PBL collected from RA patients	In the early stage of RA, sCD83 level is high in plasma but in the chronic stage, sCD83 level is high in SF rather than plasma.	[109]
	2018	Examined PBL samples from RA patients	CD83 expression in B lymphocytes performs many regulatory roles in risk variants of RA disease.	[110,111]
	2019	Analyzed the blood, hind paw, bone marrow cells, bone marrow extracted monocytes, synovial and, LN cells of mice infected with antigen-induced arthritis	sCD83 gave an attractive possibility for controlling the long-term immune response and inflammatory reduction in autoimmune RA.	[112]
	2020	PBL collected from RA patients	Higher expression levels of CD83 were observed in RA patients who were treated with an antirheumatic drug known as abatacept.	[113]

### 4.3. Atopic Dermatitis

Atopic dermatitis (AD) is considered a complicated, persistent, and inflammatory skin disease [114]. Although the individual route of AD is not completely understood yet, epidermal barrier disturbance, genetic predisposition, and an imbalanced immune system are some aspects of AD [115]. AD is characterized by dry and sensitive skin with localized or disperse eczematous lesions, commonly accompanied by an extreme itching sensation. The clinical heterogeneous phenotype of AD varies depending on age, ethnic background, and severity [116]. Interestingly, autoreactivity has a potential role in the etiology of AD. The severity score of AD has been related to IgE levels, allergen sensitization, and the occurrence of autoreactive IgE [117,118]. Recent studies have revealed that altered DC functions were detected in AD patients, demonstrating that DCs are crucial in the emergence of skin inflammation [119]. Additionally, DCs present many TLRs for detecting danger signals [120]. Its stimulation can result in DC maturation and activation, which is indicated by the expression of costimulatory molecules and the release of cytokines [121,122]. Among co-stimulatory molecules, CD83 expression is greater and frequently arranged in distinct dermal clusters in AD [97]. In a normal epidermis, Langerhans cells (LCs) constitute the majority of the DCs, although AD is also colonized with inflammatory dendritic epidermal cells (IDECs) [123]. LCs from healthy and AD skin exhibit similar CD83 levels, whereas freshly separated IDECs display a CD83 marker expression pattern more consistent with matDCs. Freshly separated IDECs and LCs from AD patients show less TLR2 expression, although only IDECs from AD skin are highly matured in an equilibrium state. These results lend greater credence to the hypothesis that AD patients are much more vulnerable to cutaneal infections due to aberrant TLR2 expression, a loss of downstream signaling cascade in epidermal DC, and elevations in levels of DC markers such as CD83 and other T cell markers [99,124]. Recent investigations have demonstrated that monocytes as precursors of DCs can quickly penetrate inflamed tissues, drive DC differentiation, and lead to the pathophysiology of AD [125]. Moreover, MoDCs obtained from AD patients increased the expression of CD83 less significantly than healthy control MoDCs after anti-CD40 Ab stimulation [126]. Transglutaminase-3 (TGase-3) is a member of the transglutaminase family and is expressed in the granular and spinous layers of the epidermis [127]. TGase3 expression is noticeably increased in many skin inflammations. Additionally, inhibiting TGase3 can reduce skin inflammation in a transgenic model of MC903-inserted AD. However, it is unclear exactly how TGase3 contributes to AD pathogenesis. Su et al. [98] also conducted a comprehensive experiment on MoDC culture stimulated with TGase-3 and found higher expression of CD83 and other DC markers, collectively indicating that TGase3 might play a significant role as a therapeutic for the prevention of AD. Previous studies have mentioned that DCs are among the primary regulators for the beginning of allergic reactions in AD patients, being aggressively activated by epithelial-cell-derived thymic stromal lymphopoietin (TSLP), a cytokine that can dramatically upregulate the expression of CD83. Some other DC markers are predominantly expressed by epithelial cells, particularly keratinocytes obtained from AD patients [96]. When allergic reactions first appear, the peroxisome proliferator-activated receptor (PPAR)γ, which operates as a down-regulator in various immune cells, can inhibit the DCs maturations by suppressing the expression of CD83 and other co-stimulatory receptors in TSLP-stimulated DCs derived from mice. There is evidence that PPARγ is one of the helpful suppressors of initial AD events [128]. Therefore, identifying the expressions of CD83 markers could certainly provide valuable data for AD treatment.

### 4.4. Behçet’s Disease

Behçet’s disease (BD), an inflammatory condition with several manifestations and an unidentified etiology, is characterized by eye, oral aphthae, ulcers, skin infections, vascular, articular, gastrointestinal, neurologic, and pulmonary involvement [129]. BD shares some common characteristics with auto-inflammatory and autoimmune disorders. Although the actual cause of BD is unknown, some environmental factors, genetic susceptibility [130], and immunological irregularity [131], that have been identified as playing crucial roles in BD development. T cell abnormalities are also connected to BD [131] and might be activated by DCs. Additionally, co-stimulatory markers such as major MHC-II, CD86, CD80, CD83, and CD40 expressed on DCs can elevate T cell receptor signaling, which can result in T cell development and activation [132,133]. MHC class II, peptides, and costimulatory molecules are all accumulated by DCs as they mature. Relatively high expression of mature biomarkers on DCs may signal the presence of BD symptoms. Among matDC biomarkers, CD83 is one of the matDC biomarkers that is essential for the immune response and also functions as an activation signal [10]. Intriguingly, the occurrence of mature CD83 expressions on the peripheral blood leukocytes (PBL) is noticeably greater in mice with BD manifestations that can contribute to BD progression [100]. Furthermore, stress can accelerate DC maturation by enhancing activation markers. Among all stresses, noise stress has a significant role in BD, and it may make symptoms worse by increasing the frequency of CD83+ molecules [101]. BD patients have a unique dysbiosis in gut microbiota with much less butyrate synthesis [134]. Approximately 13% of such gut microbiota are made up of *Eubacterium rectale* (*E. rectale*), which is necessary for butyrate synthesis [135]. Treatment with *E. rectale* can meliorate BD symptoms in HSV-1 infected mouse models by modulating DC activation in addition to downregulating CD83+ molecules [136].

Undoubtedly, BD can cause anterior, and posterior uveitis and retinal vasculitis. Recently, it has been discovered that matDCs can promote the inflammatory condition of posterior uveitis that develops inside the choroid under uveitic conditions. Furthermore, adoptively transferring matDCs primed with uveitogenic antigens may cause experimental autoimmune uveitis (EAU) [137,138]. sCD83 administration can decrease disease severity in EAU by inhibiting filamentous-actin-dependent calcium signaling cascade in DCs and restricting DCs-mediated CD4+ T cell activation. Likewise, sCD83 induction in EAU can reduce inflammation by producing NK cells [139] and inducing tolerogenic (tol) DCs. Similarly, teriflunomide, a metabolite that can be used to treat uveitis, can reduce DCs maturation and also express fewer CD83 and CD86 surface receptors in an EAU-teriflunomide-treated model [140]. Treg cells have now been thoroughly studied in relation to autoimmune diseases [141,142]. Remarkably, Treg cell differentiation during activation depends on CD83. However, there is conflicting information regarding Treg cell frequency in BD patients [143]. In certain studies, Treg cell frequency in PBL and CSF is higher [144], whilst in other studies, the frequency is shown to be lower [145]. Additionally, Treg cells that no longer express CD83 can downregulate specific Treg cell differentiation markers and induce an inflammatory phenotype [23]. Another unifying manifestation of BD is a skin pathergy-reaction (SPR), a small trauma in a skin tissue that involves epithelial rupture. Activated DCs are primarily responsible for CD83 expressions inside the dermis, with considerable increases observed in sections taken from SPR areas compared to sections taken from healthy individuals. The epidermal–dermal border is the main location for these massive CD83 cells, which is indicative of freshly triggered LCs migration out from the epidermis further into the dermal layer. Similarly, CD83 mRNA expression is also considerably higher in SPR tissue samples than in tissue biopsies from healthy subjects. MatDCs influx more frequently in the skin of BD patients with SPR [146]. Oral ulcers in BD patients are the first clinical manifestation, and LCs such as APCs play a significant role in various oral pathologic conditions. LCs can move into the lamina propria, where they grow into mature CD83+ DCs [147]. CD4+ T cell production, which is important in the pathophysiology of BD, is hampered when CD83 expression is disturbed [131,148]. Thus, CD83 might be extremely important for controlling the inflammatory response and preserving immunological balance in BD.

### 4.5. Multiple Sclerosis

Multiple sclerosis (MS) is a demyelinating, disabling T cell-intermediated autoimmune and chronic neuroinflammatory disorder of the central nervous system (CNS). It generally affects young adults [149,150]. MS seems to be a worldwide disease that is progressively increasing. The fundamental cause of MS and the mechanisms causing such an increase remain unknown. Complicated environment and gene interactions might play a crucial role [151]. Immune cell infiltration and aggregation in the CNS that target myelin are linked to brain dysfunction in MS [152]. Recent research has suggested that DCs are innate immune cells that can perform a pathogenic role in MS. Compared to healthy individuals, MS patients have an abundance of DCs in their brain lesions with a distinct phenotypic and/or function. Therefore, DCs have the potential to pathologically affect how auto-reactive T and B cells operate as effectors [153]. By releasing a variety of cytokines, DCs can direct naïve T cells toward differentiating into numerous subtypes of T helper (TH) cells [154]. Furthermore, studies have suggested that CD83 marker shows modest levels of expression in matDCs and tolDCs produced by MS patients [102]. However, tolDCs are considered an immunotherapy for MS [155]. Conversely, the concentration of the sCD83 form is also higher in MS patients [104]. Another approach is specified in the experimental autoimmune encephalomyelitis (EAE) model, a multiple sclerosis (MS) model. A recombinant soluble CD83 (rsCD83) protein that exploits the inhibition of CD83 was found to be effective in this model. Therefore, EAE can be successfully stopped by intraperitoneal administration of rsCD83, which may result in a reduction in T cell cytokines, particularly IL-10, IL-4, IL-2, and IFN-γ [156].

It has been recently demonstrated that Treg cells can express CD83, which is crucial for the stability and differentiation of Treg cells [23]. When EAE is induced in a bacterial artificial chromosome transgenic mouse model expressing humanized CD83, disease manifestations are noticeably improved, and Treg cell activity is increased noticeably in the CNS as compared to wild-type animals. Furthermore, CD83-transduced T cells can suppress the effector stage of the paralytic EAE symptomatic model [24]. Moreover, IDO, an enzyme in the kynurenine pathway (KP), can function as an immunoregulator with the help of various mechanisms, either independently or in conjunction with sCD83. IDO expressions can dramatically elevate a number of disease processes and contribute significantly to the metabolic immunoregulatory control of autoimmune [157] and chronic inflammatory [158] disorders through a variety of mechanisms. The neuro-immunomodulation axis, sCD83-IDO-KP, has significant effects on the emergence of several neurological diseases such as MS [159]. By fostering this process, IDO can suppress the activation of T cells by promoting Treg cell proliferation in EAE [157], thus leading to the development of immunological tolerance. The immunological control of Tregs is reinforced by IDO through feedback effects as a result of IFN-γ production stimulated by Tregs [160]. These results point to a remarkable function for CD83 as a powerful immune-regulating marker in MS and EAE, in addition to its possible role in the development of Treg-targeted therapies for autoimmune disorders [105]. Further research has found that the CNS of MS patients offers a B cell-favorable environment, allowing B cells to impact MS development via a variety of mechanisms [161]. Interestingly, a higher frequency of CD83+ B cells has been observed in secondary progressive MS patients [103]. Vitamin D3 has a strong beneficial effect on MS that is related to DCs. In particular, it can inhibit mature markers such as CD83 [162]. A drug called ethyl pyruvate (EP) has been used to treat MS and improve the EAE model [163]. EP can also produce tolDCs. These EP-treated DCs can reduce CD83 and other markers needed for the activation of T cells. It can also inhibit proliferation and alter cytokine production [164]. Therefore, the identification of CD83 marker expression would certainly provide valuable information for the management of MS [159].

### 4.6. Rheumatoid Arthritis

Rheumatoid arthritis (RA) is an inflammatory immune-mediated disorder in which several immune cells and signaling processes are dysfunctional, causing a maladaptive tissue-degenerative process that damages organs, especially joints [165,166]. RA is manifested as infiltration of synovial compartments with different varieties of immune cells, particularly DCs, monocytes, T cells, B cells, NK cells, and neutrophils. DCs involvement in RA has been extensively investigated [167]. Strikingly, patients with RA have higher levels of DC biomarkers, particularly CD83, CD80, and CD86, in their synovial fluid (SF) and synovial tissue (ST) [106]. Interestingly, CD83 as a percentage of whole DCs was low in ST taken from RA and psoriatic arthritis patients [108]. Furthermore, Treg cells are essential for reducing inflammation, preventing bone loss, and suppressing osteoclast differentiation in arthritic joints [168]. Notably, sCD83 can promote Treg cell formation and expansion [43], which not only inhibits persistently autoimmune responses but also activates intrinsic mechanisms that cause inflammatory processes to resolve in arthritis.

IDO functions as a signaling protein to activate TGF-β expression [169], and this function can be applied for its beneficial role in RA. Furthermore, in the antigen-induced arthritis (AIA) model, IDO1 can exert anti-inflammatory activity in pDCs upon IFN-α treatment [170]. In a similar model, sCD83 treatment can resolve inflammation in an IDO1/TGF-β-dependent way, indicating the participation of IDO1 signaling action [171] in RA. Additionally, the immunomodulatory effect of sCD83 is observed in arthritis by upregulating TGF-β and IDO1 and enhancing direct osteoclastogenesis impairment [112]. Moreover, SFs taken from RA patients contain a relatively high amount of sCD83, which raises the possibility that they may contribute to the pathophysiology of RA [107]. Furthermore, sCD83 has no the ability to differentiate the osteoclasts directly; instead, it modulates the TLR-4 axis, the TLR4/MD2 complex present on monocytes [42], and the receptor of sCD83, and then monocytes differentiate into osteoclasts [172] but still contain TLR4 upon their surface [173]. It is feasible that sCD83 might be involved in osteoclastogenesis through this pathway. Moreover, it appears logical to suppose that measuring sCD83 in the serum of patients with the inflammatory disorder may serve as an early predictive autoimmunity marker to alternatively detect and cure more extreme symptoms. Significantly, early stages of RA that are unaffected by anti-TNF-α antibody treatment have higher sCD83 expressions in plasma. However, in cases of chronic arthritis, sCD83 expressions are found to be higher in SF than in plasma [109]. Recently, according to Royzman et al. [174], sCD83 has been demonstrated to represent a therapeutic target in an arthritis mouse model by downregulating various osteoclast-inducing factors and also enhancing metallothionein family members that are reduced in the arthritis mouse model. As we know about the two subtypes of DCs, mDCs, and pDCs [175], CD83 expressions are similar between RA and healthy subjects at all stages of pDCs maturation but are increased on RA mDCs [176]. CD83 molecular expression in B lymphocytes may play many regulatory roles in risk variants of RA disease [110,111]. In addition, the latest study conducted on RA patients who responded to treatment with the anti-rheumatic drug abatacept showed higher expression levels of CD83 in their blood [113]. A recent publication revealed that an anti-mouse CD83 monoclonal antibody (DCR-5) targets CD83 and causes the depletion of pre-existing DCs while increasing levels of Treg and regulatory DCs in a mouse model of RA [177]. Thus, CD83 might be essential for controlling inflammatory processes and maintaining immunological tolerance in RA.

**Table 2:** Potential anti-CD83 therapeutics in inflammatory disorders.

Diseases	Published Year	Treatment Material	Model	Potential Mechanism	Ref.
IBD	2014	sCD83	Mice	sCD83 ameliorates colitis in the murine model by drastically reducing the inflammatory cytokines in the colon.	[70]
	2015	Murine CD83+ T cells	Mice	In vivo, CD83+ T cells inhibit colitis symptoms in a colitis model by suppressing inflammatory cytokines and the release of sCD83.	[21]
SLE	2008	Curcumin	Human	Decrease CD83 expressions by inhibiting the proliferation of CD4+ T cells and DC migration and limiting the levels of the chemokines.	[93]
	2013	Human sCD83	Mice	sCD83 producing its autoantibodies intervene with autoantibodies that are already present in SLE, thus acting as an immunomodulator.	[92]
AD	2011	Synthetic PPARγ ligands	Mice	Due to its ability to suppress CD83 and other matDC markers’ expression, PPAR may display a significant therapeutic approach in the initial stages of AD.	[128]
BD	2019	Abatacept, CD83 siRNA	Mice	Decrease mature CD83 in BD mice.	[100]
	2021	*E. rectale*, (*E. rectale* + colchicine)	Mice	Induction of *E. rectale* into BD mice lessens the CD83+ levels in BD mice.	[136]
MS	2004	A soluble form of hCD83ext	Mice	Soluble forms of hCD83ext act as immunosuppressive agents that prevent paralysis related to EAE.	[156]
	2010	Vitamin D3	Human	Vitamin D3 has beneficial effects related to its impact on DCs in MS patients particularly inhibiting mature CD83 markers.	[162]
	2015, 2019	Ethyl pyruvate	Human and rats	EP is used for MS treatment and also ameliorates EAE model by reducing the CD83 expressions using EP-treated DCs.	[163,164]
RA	2022	sCD83	Mice	sCD83 acts as a therapeutic target in an arthritis model by downregulating the various osteoclastogenic factors, and also enhancing the metallothionein family members that are reduced in the arthritis mice model.	[174]
	2022	Anti-Mouse CD83 Monoclonal Antibody (DCR-5)	Mice	Target CD83, causing in depletion of conventional DCs and increasing the expression levels of Treg cells and regulatory DCs in RA model.	[177]

## 5. Conclusions

CD83 can function as a regulatory molecule that plays a pivotal role in autoimmunity and autoinflammation through various mechanisms to reduce inappropriate immunological responses. Excessive recruitment of CD83 during inflammatory processes indicates an important function of CD83 in the immune system. On the other hand, modified forms of CD83, with different functions of sCD83 and mCD83, may play a role in preventing inflammatory diseases. However, to date, there are still conflicting reports regarding the protective and stimulatory roles of CD83 in resolving diverse inflammatory responses. Therefore, further knowledge is needed to fully understand the functions of CD83 and its precise regulatory mechanisms that may help unravel the understanding of several diseases. In addition, the application of modified CD83 molecules could be a promising strategy for treating inflammatory and autoimmune diseases. In this regard, further in vitro and in vivo studies and prospective trials are needed.

## Data Availability

Not applicable.

## Abbreviations

Symbol	Description
AD	Atopic dermatitis
BD	Behçet’s disease
cKO	Conditional knockout
DCs	Dendritic Cells
EAE	Experimental autoimmune encephalomyelitis
ELISA	Enzyme-Linked Immunosorbent Assay
HSV	Herpes simplex virus
IDO	Indoleamine 2,3-dioxygenase
IDECs	Inflammatory dendritic epidermal cells
iDCs	Immature dendritic cells
IBD	Inflammatory bowel disease
LCs	Langerhans cells
mbCD83	Membrane-bound CD83
MHC	Major-histocompatibility-complex
MARCH	Membrane-associated RING-CH
matDCs	Mature dendritic cells
mDCs	Myeloid dendritic cells
MS	Multiple sclerosis
pDCs	Plasmacytoid dendritic cells
PBL	Peripheral blood leukocyte
PBMCs	Peripheral blood mononuclear cells
PMQs	Peritoneal macrophages
PsA	Psoriatic arthritis
qRT-PCR	Quantitative reverse transcription polymerase chain reaction
RA	Rheumatoid arthritis
sCD83	Soluble CD83
SLE	Systemic lupus erythematosus
SF	Synovial fluid
TEC	Thymic-epithelial cell
tolDCs	Tolerogenic dendritic cells
TNF	Tumor necrosis factor
